# Cell-free DNA profiles of dermatomyositis and its potential role in discriminating phenotypes

**DOI:** 10.3389/fimmu.2025.1605121

**Published:** 2025-06-17

**Authors:** Zhuang-li Tang, Peng-yu Chen, Heng Zhang, Hua-li Cao, Ru Dai, Yu-chen Lou, Yan-hong Sun, Yuan Zhou, Xue-yan Chen, Mei-jie Zhang, Ya-qi Wang, Xiao-yong Man

**Affiliations:** Department of Dermatology, Second Affiliated Hospital of Zhejiang University, Hangzhou, China

**Keywords:** cell-free DNA, dermatomyositis, fragmentation, 5’ end-motif, genetic variance

## Abstract

**Background:**

Cell-free DNA (cfDNA) functions in the early-detection and monitoring of autoimmune diseases including systemic lupus erythematosus and rheumatoid arthritis. However, investigations into cfDNA profiles in dermatomyositis and their potential clinical implications remain scarce.

**Objectives:**

To explore the overall landscape of cfDNA profiles in dermatomyositis and investigate potential roles in discriminating subtypes.

**Methods:**

Following informed consent, 24 treatment-naïve patients diagnosed with dermatomyositis and 16 healthy controls were enrolled. We examined cfDNA concentrations, fragment distribution patterns, 5’-end motif frequencies and genetic variation profiles in all participants and studied potential correlation with laboratory parameters. Moreover, intergroup differences of cfDNA profiles among patients and potential correlation between extracellular DNases levels and cfDNA were investigated.

**Results:**

Compared to healthy controls, dermatomyositis patients exhibited elevated cfDNA concentrations, with significantly longer cfDNA fragments, primarily centered around 180–360 bp; nonetheless, no correlation was witnessed between lab parameters and cfDNA levels. The A-end predominated the 5’-end motif, whereas the C-end was underrepresented, contrasting with the patterns observed in healthy controls. In addition, genetic variations in several genes, including *PDE4DIP* and *BRCA2*, were commonly detected in cfDNA from dermatomyositis patients. Notably, end-motif profiles and cfDNA fragment length exhibited variations between anti-transcription intermediary factor 1-gamma positive patients with and without malignancies. However, owing to limited sample size, we failed to draw conclusions regarding extracellular DNase levels.

**Conclusions:**

This study presents the first comprehensive depiction of cfDNA profiles in patients with dermatomyositis. Furthermore, cfDNA features exhibit variability across some sub-phenotypes and may serve as discriminatory indices. Finally, potential involvement of extracellular DNases in cfDNA profiles in dermatomyositis shall be further investigated.

## Introduction

Dermatomyositis is a severe condition that can result in significant morbidity and mortality. However, many patients remain undiagnosed based on current diagnostic and classification criteria (1). The delay between initial clinical manifestations and definitive diagnosis is common, with a median delay of 15.5 months (2). Furthermore, although the categories of dermatomyositis are far accurate on the basis of myositis-specific antibodies (MSAs) and other laboratory parameters, heterogeneity remains a significant concern in certain phenotypes, such as concomitant malignant tumors, if any, in dermatomyositis with anti-transcription intermediary factor 1-gamma (TIF1g) antibody (3).

Cell-free DNA (cfDNA) represents a small fraction of the total DNA pool that circulates freely in the bloodstream under both normal and pathological conditions. Altered cfDNA profiles have been observed in numerous autoimmune diseases, including systemic lupus erythematosus and rheumatoid arthritis (4). Recent studies have demonstrated the potential of cfDNA profiles in assisting patient stratification, monitoring therapeutic responses, and predicting disease progression (5).

Prior research has exhibited the potential clinical value of cfDNA levels in monitoring disease severity indices of dermatomyositis including the cutaneous dermatomyositis disease area and severity index (CDASI) and myositis disease activity assessment visual analogue scale (MYOACT) (6, 7). However, these studies have primarily focused on specific phenotypes of dermatomyositis, leaving the overall cfDNA profile of the disease still poorly understood.

The present study aims to explore the overall plasma cfDNA levels, fragmentation profiles and genetic variances landscapes of patients with dermatomyositis. We also seek to investigate correlation between cfDNA and laboratory parameters and compare cfDNA profiles between anti-TIF1g antibody-positive dermatomyositis patients with and without malignancies, as well as other MSA phenotypes, to assess its potential in distinguishing phenotypes. Additionally, we aim to elucidate the role of extracellular DNases, i.e., DNase-1 and DNase1l3, in the cleavage and fragments formation of cfDNA fragments, and their potential correlation with phenotypic variations.

## Patients and methods

### Patient selection and sample collection

This study enrolled 24 treatment-naïve patients diagnosed with dermatomyositis according to the 2017 EULAR/ACR classification criteria without any active infection or inflammation aside from dermatomyositis and its associated conditions, from the inpatient ward of dermatology, and 16 healthy controls from physical examination center of the Second Affiliated Hospital of Zhejiang University (Hangzhou, China) from 2022 to 2024. Ethical approval was granted by the Ethics Committee of the Second Affiliated Hospital of Zhejiang University (IR2023343). All participants have signed written informed consent. The study adhered to the principles of the Declaration of Helsinki.

Cell-free plasma samples were collected from patients once diagnosed. Patients were subdivided into three subgroups based on MSAs and cancer history: Subgroup 1 (positive anti-TIF1g antibody with tumor history, TWT; 6 patients), Subgroup 2 (positive anti-TIF1g antibody without any malignant tumor up to the most recent follow-up in Nov, 2024, TOT; 5 patients), and Subgroup 3 (other MSAs group, OM; 13 patients). Peripheral blood samples were collected using Streck tubes and were centrifuged within 24h of collection at 350×g for 10min at room temperature and the plasma was further centrifuged again at 3000×g for 15min at 4°C. All plasma samples were collected and stored at -80°C.

### cfDNA quantification in plasma and next-generation sequencing

cfDNA extraction and quantification were performed using a standard commercial kit following the manufacturer’s instructions (Plasma cell-free DNA isolation Kit, BunnyMag, Cat. No. TQ01BT0100). The cfDNA concentration was measured by Qubit dsDNA HS Assay Kit (Q32854, Invitrogen). Among them, 10 plasma samples meeting the sequencing requirements (total cfDNA > 20 ng/ml) were additionally processed for library preparation and 650 genes panel exome sequencing to evaluate the molecular characteristics of cell-free DNA. The libraries were constructed using the Hieff NGS Ultima Pro DNA Library Prep Kit for Illumina (Yeasen, Cat. No. 12201ES24). Exome capture was performed using SureSelectXT Human All Exon V6 (Agilent) technology. The sequencing was carried out on the Illumina NovaSeqXplus PE150 platform (150 bp × 2 paired- end format).

### Sequencing data processing and alignment

Quality control was performed with Fastp (v0.20.0) (8) to trim the sequencing adaptor and eliminate low-quality reads. Subsequently, the cleaned reads were aligned to the human reference genome (GRCh37/HG19) using Burrows Wheeler Aligner (BWA, v0.7.17) as previously described. PCR duplications were identified using Picard-tools (v4.1.1.0) and subsequently removed. Reads with low mapping quality (< 30), multiple alignments, or more than five mismatches were filtered out. Only paired end reads with proper mapping orientations and an insert size below 600 bp were retained for downstream analysis.

### Detection and annotation of genetic variances

The SNPs and small fragment insertions/deletions were detected by Mutect2 software from the Genome Analysis Toolkit (GATK, v4.1.1.0), meanwhile, detected VCF files were annotated by Annovar (v201804). The reference database includes COSMIC database, dbSNP Database, 1000 Genomes Project, etc. Annotation included variant location, type, and conservation predictions.

### Quantitative analysis of cfDNA

Quantitative levels of cfDNA were measured in haploid genome equivalents per milliliter (hGE/mL), calculated by multiplying the total cfDNA concentration by the mean allele fraction of somatic mutations.

### 5’ End-motif analysis of cfDNA fragments

End motifs were determined as the terminal nucleotide sequences at each 5’ fragment end of cfDNA molecules. The base content proportions of the end motifs were calculated at each position. Motifs were grouped based on fragment size, and the frequency of each motif was determined for each fragment size.

### Quantification of the level of DNase-1 and DNase1l3 in plasma

DNase-1 and DNase1l3 levels in plasma were measured using human DNase-I ELISA kit (Cusabio, CSB-E09068h) and human DNASE1L3 ELISA kit (Cusabio, CSB-EL007052HU) according to the manufacturer’s protocol.

### Statistical analysis

Differences between groups for continuous variables were assessed using the Mann-Whitney U-test, Wilcoxon test, and Kruskal-Wallis-test. The difference in categorical variables between groups was examined by the Chi-square test or Fisher’s exact test. Spearman co-efficiency analysis was adopted for correlation analysis. Statistical significance was defined as a p-value < 0.05. All statistical analysis was conducted by Prism10 for macOS (version 10.3.1).

## Results

### Demographic profiles of dermatomyositis patients

**Figure 1:**
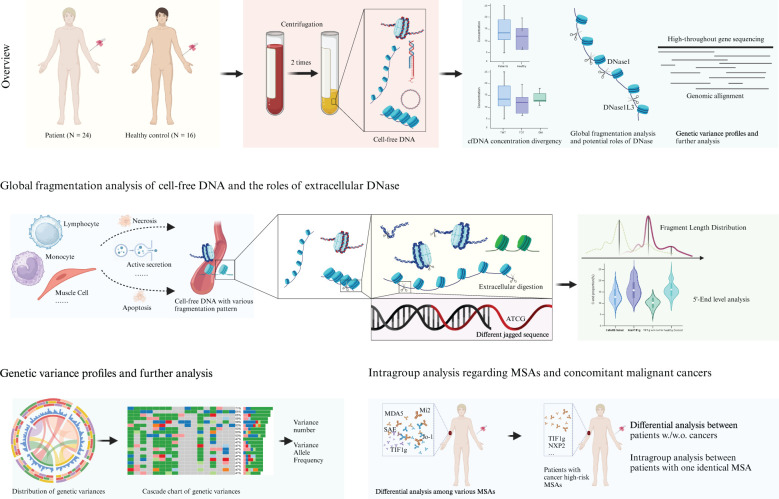
Schematic algorithm of the study.

A total of 24 adult treatment-naïve Chinese patients diagnosed with dermatomyositis according to the EULAR/ACR classification criteria (2017) were enrolled from 2022 to 2024. Of these patients, 11 were anti-TIF1g antibodies positive, with 6 having a cancer diagnosis and 5 without cancer; while the MSAs of the remaining 13 patients were not TIF1g. Demographic and laboratory data are presented in **Table 1**, and **Figure 1** provides an overview of the study.

**Table 1:** Demographic, laboratory information of enrolled patients and cfDNA sequencing parameters.

Patient Number	Gender	Age	Subgroup^1^	MSAs/MAAs^2^	Concomitant tumors	Time interval^3^	NLR^4^	ESR(mm/h)^5^	CK(U/L)^6^	Ferritin(μg/L)	cfDNA(ng/μl) ^7^	Mapping Fraction(%)	PCR duplicate Fraction(%)	Average depth(rmdup)	Coverage (>=10x)(%)	Raw_Bases(G)	Clean_Bases(G)	Q20(%)	Q30(%)	GC content(%)	PCR Dupulication(%)
1	female	57	TOT	TIF1γ			4.01	2	165	43.1	0.48	99.74	75.33	1703.9	99.95	41.13	40.49	97.807	93.79	46.951	48.68
2	male	75	TWT	TIF1γ	stomach cancer	-2 years	6.77	3	100	17.5	0.28	99.68	64.36	1596.1	99.71	27.412	26.957	97.754	93.655	46.119	33.434
3	female	58	TWT	TIF1γ	cervical cancer	3 months	4.60	17	59	113.9	0.24	99.52	55.81	1192	99.58	26.918	26.439	97.551	93.159	44.782	29.64
4	female	31	OM	MDA5			3.16	15	348	357.1	0.35	99.6	55.45	2038.6	99.72	24.199	23.712	97.528	93.249	45.794	26.566
5	female	49	OM	Mi2			3.00	2	49	68.9	0.35	99.7	67.8	2642.9	99.68	39.578	38.95	97.834	93.92	46.912	39.719
6	female	67	TWT	TIF1γ	breast cancer	-15 years	12.16	16	469	N/A	1.14	99.67	50.73	4474.9	99.87	39.268	38.592	97.927	94.273	47.559	21.358
7	male	52	TWT	TIF1γ	esophageal cancer	6 months	8.45	52	56	363.6	0.18	99.67	71.69	1505.7	99.68	40.824	40.157	97.841	94.065	47.673	43.971
8	male	73	TWT	TIF1γ	gallbladder cancer	6 months	10.51	2	128	393	0.25	99.62	68.18	1433.9	99.86	33.223	32.71	97.784	93.928	45.995	42.955
9	female	75	OM	SAE			3.03	46	68	N/A	1.58	99.7	49.65	4300.1	99.82	36.458	35.946	97.829	93.987	48.578	18.727
10	female	51	OM	Ro52			2.65	7	590	405.8	0.54	99.74	48.9	3061.6	99.85	23.234	22.841	98.051	94.422	48.177	23.034
11	female	66	OM	Mi2			9.96	15	420	173.9	1.56	99.71	46.86	4563.6	99.66	36.711	36.168	97.79	93.793	48.048	14.926
12	male	56	OM	NXP2			18.92	39	577	598.4	0.60	99.71	48.95	4225.4	99.55	37.15	36.637	97.719	93.639	48.43	17.701
13	male	55	OM	PM-SCL100			2.50	2	210	226.9	0.15	99.67	64.9	1283.1	99.7	22.961	22.455	96.738	91.787	46.045	30.782
14	female	51	TOT	TIF1γ			7.27	18	41	62.6	0.41	99.68	52.61	2881.9	99.5	32.448	31.998	97.529	93.184	48.016	21.121
15	female	58	OM	MDA5			2.40	42	99	603.9	2.70	99.69	40.68	3912.7	99.92	28.564	28.162	97.394	92.926	47.151	11.979
16	female	20	TOT	TIF1γ			3.34	2	20	N/A	0.31	99.62	50.43	1968.7	99.74	22.99	22.598	98.179	94.882	48.334	17.177
17	male	60	OM	MDA5			4.79	19	796	1783.6	1.25	99.67	36.22	4082.6	99.65	28.231	27.696	98.423	95.506	48.351	6.112
18	female	45	OM	Mi2			8.03	23	4275	N/A	0.32	99.67	60.1	2521.9	99.67	41.593	40.468	98.24	94.938	47.858	13.602
19	female	65	TOT	TIF1γ			4.37	2	35	N/A	0.99	99.74	46.87	4133.5	99.91	32.918	32.535	98.325	95.14	48.109	9.465
20	female	57	OM	PM-SCL100			13.88	29	52	215.5	0.40	99.83	60.84	3957.2	99.74	42.254	41.474	98.416	95.379	47.947	14.24
21	male	71	OM	MDA5			6.05	54	96	1480.6	0.19	99.81	84.48	595.4	99.73	29.095	28.608	98.49	95.572	46.67	50.76
22	female	52	TOT	TIF1γ			2.64	4	38	N/A	0.34	99.82	57.5	4359.1	99.72	40.728	39.857	98.318	95.137	48.161	11.299
23	male	31	OM	MDA5			3.22	3	58	505.2	0.52	99.74	53.06	5653.2	99.61	40.882	40.008	98.022	94.576	48.083	6.697
24	female	50	TWT	TIF1γ	ovarian cancer	-31 months	3.01	51	115	454.2	0.36	99.8	77.95	1234.4	99.68	34.406	33.976	98.555	95.424	47.332	39.889

^1^TWT, TIF1g positive with tumors subgroup; TOT, TIF1g positive without tumors subgroup; OM, Other myositis-specific antibodies positive subgroup.

^2^MSAs/MAAs, myositis-specific antibodies/myositis-associated antibodies. If there is no positive result of MSAs for any patient, the result of MAAs will be recorded.

^3^Time interval, the time interval between the diagnosis of dermatomyositis and concomitant tumors. Positive values indicate the tumors are confirmed after the diagnosis of dermatomyositis while negative values indicate the tumors occur prior to the diagnosis of dermatomyositis.

^4^NLR, neutrophil-to-lymphocyte ratio.

^5^ESR, erythrocyte sedimentation rate.

^6^CK, creatine kinase.

^7^cfDNA, cell-free DNA.

### Increased cfDNA concentration and possible correlation with lab parameters

**Figure 2:**
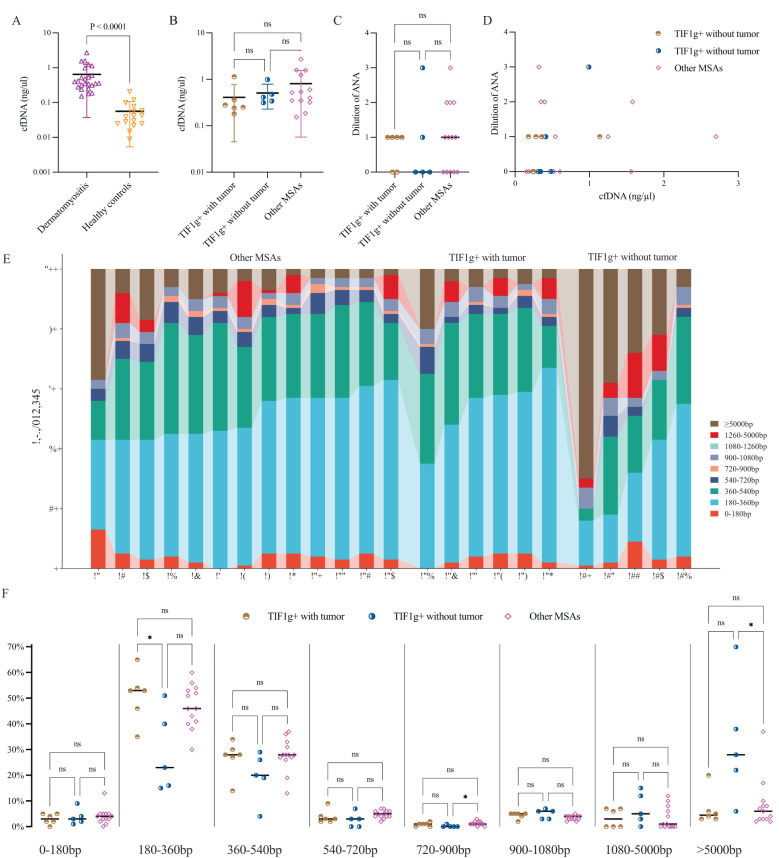
Plasma cfDNA levels, correlation with ANA and cfDNA fragment length distribution profiles in dermatomyositis. Plasma levels of cfDNA were significantly elevated in patients with dermatomyositis compared to healthy controls, but no intergroup variances were found among the subgroups **(A, B)**. ANA dilution showed no notable differences among subgroups and no clear trend was observed between ANA titers and cfDNA levels **(C, D)**. cfDNA fragments assigned to 180~360bp predominate in nearly all patients **(E)**; the intergroup variance was displayed as regards different cfDNA fragment length **(F)**. *P < 0.05. ns, not significant.

Compared to healthy controls, all enrolled patients displayed an overall higher concentration of cfDNA, with statistical significance, ranging from 0.15 ng/μl to 2.70 ng/μl (**Figure 2A**). However, no intergroup differences were identified (**Figure 2B**).

The potential pathogenicity of abnormally elevated extracellular DNA is of interest, with some studies attributing it to the formation of antinuclear antibodies (ANA) (9). Considering these findings, we explored whether cfDNA concentration correlates with ANA titers and inflammatory markers in all patients.

To streamline subsequent research, ANA titers were classified as 1:40 (dilution 1), 1:80 (dilution 2), and so on, with negative ANA as dilution 0. Generally, 14 patients were reported to be ANA positive (dilution 1 or higher), but no significant differences were found across subgroups, and no correlation between cfDNA levels and ANA titers was observed (**Figures 2C, D**).

Meanwhile, spearman co-efficiency analysis of erythrocyte sedimentation rate, ferritin and neutrophil-lymphocyte ratio with cfDNA levels showed no significant associations. Furthermore, no correlation was found between cfDNA and creatine kinase, a marker of muscle damage.

### Longer cell-free DNA fragments are documented in patients with dermatomyositis

In principle, a cfDNA molecule consists of one or more nucleosome core (146bp), H1-bound/free linker DNA segments and unbound linker DNA (10). In order to investigate the overall characteristics of cfDNA fragments, the profile of cfDNA length was roughly divided into multiples of 180bp. In healthy individuals, most cfDNA was within the nucleosome-core category, with a median length of 167 bp. In contrast, dermatomyositis patients had a predominance of cfDNA fragments between 180 and 360 bp, ranging from 15% to 65% (**Figure 2E**).

Subsequently, we compared cfDNA lengths among three subgroups using the Kruskal-Wallis-test. Significant differences were observed at 180–360 bp (P = 0.035), 720–900 bp (P = 0.043) and >5,000 bp (P = 0.027). The proportion of 180–360 bp cfDNA fragments varies significantly between the TWT and TOT subgroups, which may serve as an effective indicator of concomitant tumors in dermatomyositis patients with anti-TIF1g antibodies (**Figure 2F**).

### The end-motif landscape of cell-free DNA in dermatomyositis

**Figure 3:**
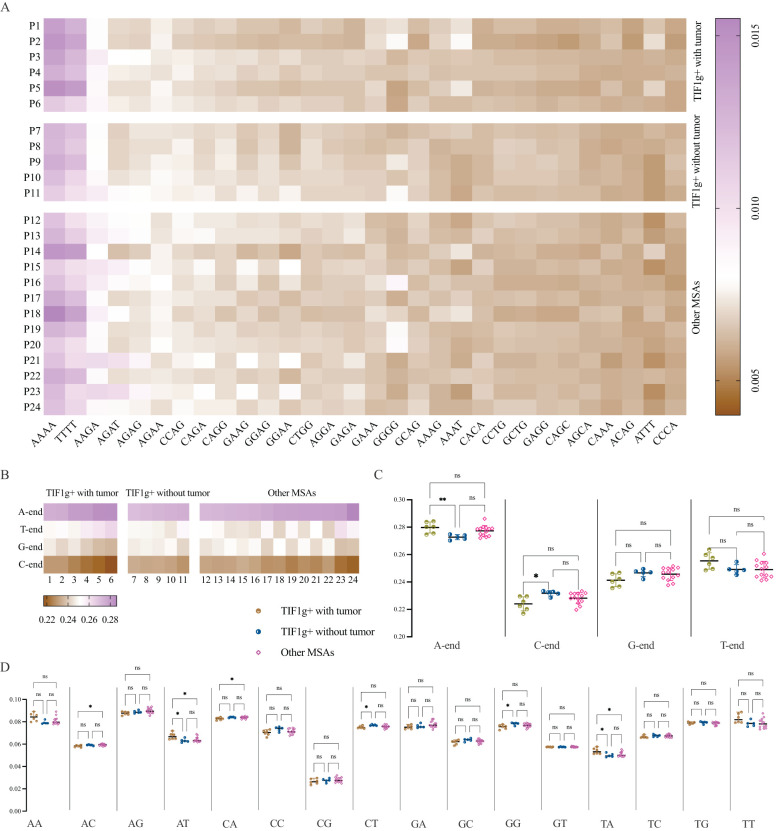
End-motif profiles of cfDNA. Heatmap of the top 30 four-mer end motifs across patient subgroups **(A)**. A-end predominates in all patients, while proportion of C-end is far lower in TWT subgroup compared to TOT subgroup **(B, C)**. The intergroup variances as regards 2-mer end-motifs **(D)**. *P < 0.05, **P < 0.01. ns, not significant.

To provide a comprehensive characterization of cfDNA fragment end profiles, the frequencies of 4-mer end-motif at each 5’ fragment end of cfDNA molecules were calculated. The 4-mer end-motif was defined as the terminal first 4-nt sequence at each 5’ fragment end of cfDNA molecules in alphabetical order, resulting in 256 categories. The top 30 4-mer end-motifs were then summarized as **Figure 3A**, with AAAA, TTTT and AAGA occupying the top three positions.

To examine broader trends, the frequency of both 1-mer and 2-mer end motifs was also calculated. The results demonstrate that the 5’ A-end predominates, while the C-end is the least prevalent end-motif in all patients with dermatomyositis enrolled in the present study (**Figure 3B**). Patients in the TWT subgroup showed higher frequencies of A-end (P = 0.0088) and lower frequencies of C-end (P = 0.0148) compared to the TOT subgroup, though no differences were found between the TOT and OM subgroups (**Figure 3C**).

Regarding 2-mer end-motifs, significant intergroup variances were observed for AC-, AT-, CA-, CT-, GG-, and TA- end-motifs frequencies. The TWT subgroup had higher frequencies of AT- and TA- motifs compared to the TOT and OM subgroups, while showing lower frequencies of CT- and GG- motifs than the TOT subgroup. It is noteworthy that the percentage of AC- and CA- end-motifs was similar between the TWT and TOT subgroups, despite overall intergroup variances (**Figure 3D**).

### Genetic variance profiles with high frequencies in patients with dermatomyositis

**Figure 4:**
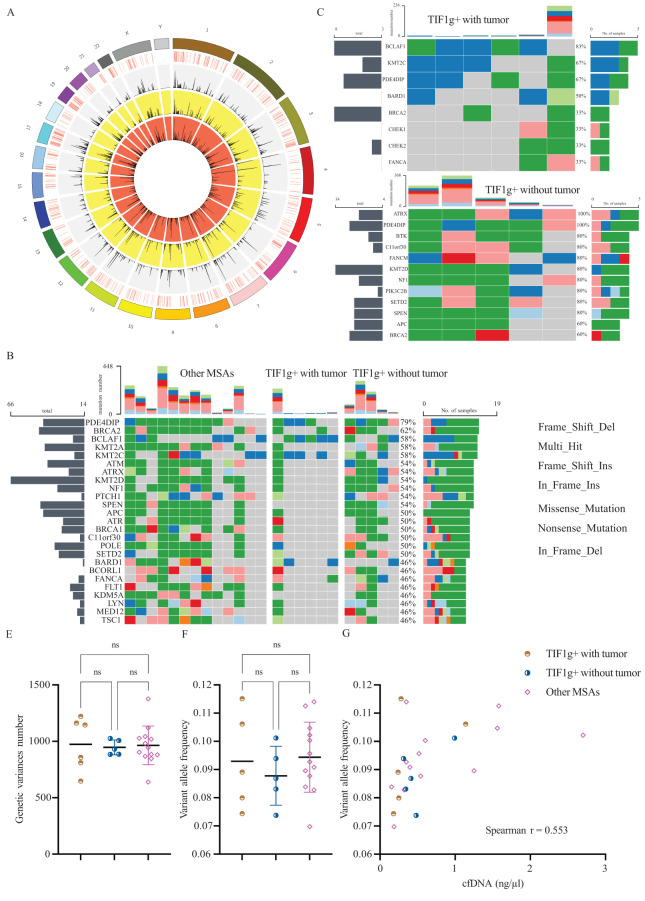
Results of high throughput cfDNA sequencing. Circos plot represents the chromosome, sequencing coverage, and the variance density from TOT, OM and TWT subgroup, respectively, from the outermost layer to the innermost **(A)**. Genetic variances for all enrolled patients **(B)**, TWT subgroup **(C)** and TOW subgroup **(D)**. Levels of genetic variances number and variant allele frequency of each subgroup **(E, F)**. Association between variant allele frequency and cfDNA levels **(G)**. ns, not significant.

The mean sequencing depth exceeded 1000x, ensuring a minimum data size of 22GB. A comprehensive summary of all the sequencing parameters is provided in **Table 1** and **Figure 4A**. Among the 24 patients enrolled, 17 genes were identified as mutated in half the patients or more (**Figure 4B**). *PDE4DIP* was identified as the most highly mutated gene (19/24), irrespective of the subgroups, followed by *BRCA2* (15/24). Furthermore, genetic variances of *BCLAF1*, *KMT2A* and *KMT2C* were detected in 14 patients. Regarding the absolute variance numbers, *KMT2D* ranked first with 66 reported variances, followed by *BRCA2* and *SPEN*. The majority of genetic variances were multi-hit combinations in patients, with frame-shift insertion genetic variances ranking highest in terms of mutation types. These findings suggest that these genes and their coding proteins may be involved in the pathogenesis of dermatomyositis.

Subsequently, we investigated differences in genetic mutations between the TWT and TOT subgroups (**Figures 4C, D**). No genetic variance of either *SPEN* or *KDM5A* was detected in the TWT subgroup, and the variance frequencies remained high in the remaining two subgroups. Furthermore, gene variances in *ATRX* were detected in all patients allocated to the TOT subgroup (5/5), but rarely in the TWT subgroup (1/6). In addition to the above finding, genetic variance frequencies of *BTK*, *FANCM*, and *PIK3C2B* differ among subgroups. This finding provides a foundation for further investigation into the potential roles of these genes.

We also examined whether there were differences in the total number of somatic mutations across subgroups. The mean number of each subgroup was found to be approximately 1,000, with no significant differences (P = 0.967, **Figure 4E**). Variant allele frequency (VAF) is a widely utilized metric for disease monitoring and prognosis in cancer. Despite the absence of intergroup divergence in terms of VAF among each subgroup (**Figure 4F**), a significant positive correlation was observed between VAF and concentration of cfDNA (Spearman r = 0.5530, P = 0.0062, **Figure 4G**). Analysis within subgroups revealed a significant positive correlation in the OM subgroup (Spearman r = 0.6272, P = 0.0245).

## Discussion

This study provides a comprehensive overview of cfDNA characteristics in dermatomyositis patients, irrespective of MSAs. Furthermore, the study has highlighted patients with positive anti-TIF1g antibody, with or without concomitant tumors, have distinct plasma cfDNA fragmentation profiles.

We observed significantly higher cfDNA levels in dermatomyositis patients compared to healthy individuals, where cfDNA levels are typically undetectable or very low (11). Observed in several autoimmune diseases such as systemic lupus erythematosus, this phenomenon is possibly attributed to tissue damage in disease status (12).

The equilibrium between cfDNA generation and clearance is crucial in both health and disease (13). Impaired clearance mechanisms may be responsible for the abnormally accumulated cfDNA (4). Potential mechanisms of cfDNA clearance *in vivo* include direct degradation by nucleases; to date, DNA fragmentation factor B (DFFB), DNase-1, and DNase1l3 are the only three nucleases that have been shown to affect cfDNA levels and/or cfDNA fragment characteristics (14). In this study, we aimed to ascertain the involvement of the extracellular nucleases, specifically DNase1l3 and DNase-1, in the cfDNA profiles of dermatomyositis patients. However, due to the variability in the concentration of cfDNA in the collected peripheral blood samples, only 27 ELISA assays were conducted on serial plasma samples from nine patients (one from TWT, four from TOT and four from the OM subgroup) and 16 assays were conducted on samples from eight healthy controls (data not shown). Owing to the very limited sample size and deteriorated power, we failed to conclude any concrete roles of DNase1l3 and DNase-1 in cfDNA patterns.

The frequencies of longer cfDNA fragments in dermatomyositis is higher than normal controls. In health states, the size of cfDNA fragments differs due to variable length of intranucleosomal linker DNA (15), with the main peak at around 166 bp (16). In disease conditions, apoptotic cells produce mononucleosomal fragments, and necrotic cells generate larger fragments exceeding 10 kb (17). Besides, DNase1l3 deficiency leads to an increased amount of longer cfDNA fragments as it targets nucleosomal DNA present in extracellular space (18). In mice models, DNase1l3 deficient individuals have higher frequencies of longer sized cfDNA, indicating the pivotal role of DNase1l3 in digesting cfDNA to nucleosomal size (19). Owing to limited sample size, whether DNase1l3 contributes to longer cfDNA fragments and other underlying mechanisms shall be further investigated.

End-motif analysis, which holds promise for precision medicine (20), revealed non-random production of cfDNA fragments (21). The linker region between core nucleosomes, rather than the DNA wrapping around the nucleosome core, is more likely to be cut (22). In contrast to our current findings, an over- and underrepresentation of C- and A-end motif fragments was documented in healthy states, corresponding with the distribution of nucleosome-occupied and open-chromatin regions (13, 23). This discrepancy may be attributed to the A-end preference seen in longer cfDNA fragments (>200 bp) (14), or the loss of typical CC-end motif preference in DNase1l3-deficient models (14, 19, 24).

Consistent with previous findings from malignant tumor patients (25), over- and underrepresentation of A- and C-end motif fragments are observed between the TWT and TOT subgroups. Nonetheless, contrary to typical short cfDNA fragments (145bp) predominance found in cancer patients (26, 27), no significant intergroup variance of ~180bp cfDNA fragments was observed between TOT and TWT. Hypomethylation in tumor cells is responsible for more alternative cleavage sites and thus leading to shortened cfDNA fragments (28). Further research on cfDNA methylation profiles in dermatomyositis is needed.

Our study also highlighted the potential involvement of specific genes in dermatomyositis. Up to now, scholars only reported somatic variances of *JAK2* and *TIF1g* (*TRIM33*) that may possibly correlate with dermatomyositis, but most of the studies were case reports or from malignancy-associated dermatomyositis (29, 30).

The limitations of this study include the small sample size and single ethnicity, necessitating further validation through larger, international multi-center studies. Meanwhile, the panel-based cfDNA sequencing may have missed some information compared to whole-exome sequencing. Additionally, due to the study design and insufficient follow-up data on patient prognosis, we could not draw conclusions about the relationship between cfDNA profiles and clinical outcomes such as concomitant symptoms, survival time, treatment response or relapse rates. Therefore, further long-term follow-up and continuous investigation are required to address these issues.

In summary, our study firstly portraits the general landscape of cfDNA in dermatomyositis, which encompass concentration, fragments distribution in length, end motifs and genetic variances panoramic picture. Besides, our findings stressed the potential utility of cfDNA fragmentomes in discriminating sub-phenotypes. However, we still have a long way to go as far more data are needed for a comprehensive model in early diagnosis, therapeutical surveillance or even phenotype discrimination. In addition, the underlying mechanism contributing to such cfDNA profiles including the patterns of extracellular DNase shall be further unraveled.

## Data Availability

The variation data reported in this paper have been deposited in the Genome Variation Map (GVM) in National Genomics Data Center, Beijing Institute of Genomics, Chinese Academy of Sciences and China National Center for Bioinformation, under accession number GVM000955. (https://bigd.big.ac.cn/gvm/getProjectDetail?Project=GVM000955).
